# A pan-specific antiserum produced by a novel immunization strategy shows a high spectrum of neutralization against neurotoxic snake venoms

**DOI:** 10.1038/s41598-020-66657-8

**Published:** 2020-07-09

**Authors:** Kavi Ratanabanangkoon, Kae Yi Tan, Kritsada Pruksaphon, Chaiya Klinpayom, José María Gutiérrez, Naeem H. Quraishi, Choo Hock Tan

**Affiliations:** 1 Department of Microbiology, Faculty of Science, Mahidol University, Rama 6 Road, Bangkok, 10400 Thailand; 2 Laboratory of Immunology, Chulabhorn Research Institute, Bangkok, 10210 Thailand; 3 Department of Molecular Medicine, Faculty of Medicine, University of Malaya, Kuala Lumpur, 50603 Malaysia; 4 Department of Microbiology, Faculty of Medicine, Chiang Mai University, Chiang Mai, 50200 Thailand; 5 Veterinary Hospital, The Veterinary and Remount Department, The Royal Thai Army, Nakorn Pathom, 73000 Thailand; 6 Instituto Clodomiro Picado, Facultad de Microbiología, Universidad de Costa Rica, San José, Costa Rica; 7 Anti Snake Venom/Anti Rabies Serology Laboratory, People’s University of Medical and Health Sciences for Women, Nawabshah, Pakistan; 8 Department of Pharmacology, Faculty of Medicine, University of Malaya, Kuala Lumpur, 50603 Malaysia

**Keywords:** Immunology, Diseases, Medical research

## Abstract

Snakebite envenomation is a neglected tropical disease of high mortality and morbidity largely due to insufficient supply of effective and affordable antivenoms. Snake antivenoms are mostly effective against the venoms used in their production. It is thus crucial that effective and affordable antivenom(s) with wide para-specificity, capable of neutralizing the venoms of a large number of snakes, be produced. Here we studied the pan-specific antiserum prepared previously by a novel immunization strategy involving the exposure of horses to a ‘diverse toxin repertoire’ consisting of 12 neurotoxic Asian snake toxin fractions/ venoms from six species. This antiserum was previously shown to exhibit wide para-specificity by neutralizing 11 homologous and 16 heterologous venoms from Asia and Africa. We now show that the antiserum can neutralize 9 out of 10 additional neurotoxic venoms. Altogether, 36 snake venoms belonging to 10 genera from 4 continents were neutralized by the antiserum. Toxin profiles previously generated using proteomic techniques of these 36 venoms identified α-neurotoxins, β-neurotoxins, and cytotoxins as predominant toxins presumably neutralized by the antiserum. The bases for the wide para-specificity of the antiserum are discussed. These findings indicate that it is feasible to generate antivenoms of wide para-specificity against elapid neurotoxic venoms from different regions in the world and raises the possibility of a universal neurotoxic antivenom. This should reduce the mortality resulting from neurotoxic snakebite envenomation.

## Introduction

Snakebite envenomation causes significant morbidity and mortality in the world, particularly in sub-Saharan Africa, Asia and Latin America, with about 2.7 million envenomings and between 81,000 and 138,000 deaths a year^1–3^. This is primarily due to a limited production and inadequate supply of effective and affordable antivenoms^4^. Snake antivenoms are specific against venoms used as immunogens, and those of closely related species; cross reaction or cross neutralization with venoms from other phylogenetically distant species is not often observed^5–8^. Thus, antivenoms are mainly used to treat envenomations by snakes that are native to a particular country or region, and generally cannot be used on a larger geographical scale, in contrast to immunoglobulins for rabies or tetanus toxin. Consequently, antivenoms are produced in relatively small volumes for local or regional use and, as a result, the cost of the product is high.

One way to overcome these problems is to produce pan-specific antivenoms that can neutralize large numbers of venoms from snakes inhabiting wide geographic areas^4^. Such antivenoms could save lives of victims where no locally made antivenom is available. Better still, if ‘universal’ antivenoms, like the equine anti-rabies or anti-tetanus antitoxin, could be produced in large volumes, the cost would be lower as a result of economies of scale. These lifesaving products would then be more affordable to poor people and health authorities in developing countries where the highest incidences of snakebites occur^9,10^. Furthermore, pan-specific antivenoms with wide para specificity can be useful in cases where the culprit snake is not identified or captured, and consequently species identification of the snake cannot be made. In this context, we have previously produced an experimental pan-specific equine antiserum that is capable of neutralizing 27 neurotoxic venoms from homologous and heterologous snake species inhabiting Asia and Africa. The antiserum was prepared using a ‘diverse toxin repertoire’ of the neurotoxic snakes as immunogen. The term ‘diverse toxin repertoire’ represents a mixture of toxin fractions of a large number of different but toxicologically related snakes. The immunization strategy is aimed at exposing the animals’ immune system to a large number of epitopes of the lethal toxins. This should result in the production of antibodies with a variety of paratopes against the diverse toxin epitopes, and consequently, exhibit wide para-specificity. These toxin fractions contained all the toxic components of the venoms, mostly presynaptic and postsynaptic neurotoxins and cytotoxins, but were devoid of the high molecular mass, highly immunogenic non-toxic proteins^11,12^. This antiserum neutralizes over two dozen homologous /heterologous elapid venoms of snakes inhabiting Asia and Africa^11^.

In the present study, we demonstrated that this pan-specific antiserum also neutralized nine additional neurotoxic venoms of elapids from Central America, Africa, and Australia, including sea snakes and sea kraits. Altogether, 36 neurotoxic venoms from 4 continents have been shown to be neutralized by the antiserum. The possible basis for the wide para-specificity observed, and its potential application for the preparation of ‘universal neurotoxic antivenom’ are proposed.

## Results and Discussion

### Neutralization of neurotoxic venoms by the pan-specific antiserum and their toxin profiles

The 10 neurotoxic venoms hereby tested are shown in Table 1. The list includes venoms of the coral snake (*Micrurus nigrocinctus*), the most medically important elapid in Central America, the yellow-lipped sea krait (*Laticauda colubrina*), and the beaked sea snake (*Hydrophis schistosus*) distributed from Australian waters to the Arabian Sea. Other venoms tested include those of the tiger snake (*Notechis scutatus)*, the king brown snake (*Pseudechis australis*) and the coastal taipan (*Oxyuranus scutellatus*), which are classified within WHO Category 1 most medically important snakes from Australia and Papua New Guinea. In addition, neutralization of venoms of the African species black mamba (*Dendroaspis polylepis*), the green mamba (*Dendroaspis angusticeps*), the western green mamba (*Dendroaspis viridis*), and the Senegalese cobra (*Naja senegalensis*) was assessed. All these snakes, except *Micrurus nigrocinctus*, are within WHO Category 1 species, i.e. ‘highly venomous snakes which are common or widespread and cause numerous snake-bites, resulting in high levels of morbidity, disability or mortality^13^. *M. nigrocinctus* is in WHO Category 2 snakes, i.e. ‘highly venomous snakes capable of causing morbidity, disability or death, for which exact epidemiological or clinical data may be lacking; and/or which are less frequently implicated but is capable of inducing fatal bites^13^.

**Table 1:** Lethality of 10 neurotoxic venoms from four different continents and the neutralizing efficacy of horse pan-specific antiserum.

	Elapid snakes	Challenge dose (number of LD_50_s)	*i.v*. LD_50_ (µg/g)^a^	ED_50_ (µl)^b^	ER_50_ (mg/ml)^c^	Potency, P (mg/ml)^d^
1	*Laticauda colubrine* (Bali, Indonesia) (Venom Supplies, Australia)	1.5	0.15^@^ (0.14-0.17)	170.16 (153.72-188.37)	0.030 (0.028-0.034)	0.010
2	*Hydrophis schistosus* (Penang, Malaysia) (Wild caught specimens)	2.5	0.07 ^#^ (0.05–0.09)	201.49 (193.30-210.03)	0.019 (0.014-0.025)	0.012
3	*Notechis scutatus* (southern Australia) (Venom Supplies, Australia)	2.5	0.09 ^&^ (0.06–0.14)	146.90 (129.05-167.21)	0.034 (0.022-0.052)	0.020
4	*Oxyuranus scutellatus* (Australia) (Venom Supplies, Australia)	2.5	0.03 (0.02-0.04)	69.78 (52.49-92.76)	0.027 (0.018-0.036)	0.016
5	*Pseudechis australis* (Australia) (Venom Supplies, Australia)	1.5	0.31 (0.24-0.40)	76.32 (68.22-85.37)	0.152 (0.118-0.197)	0.051
6	*Naja senegalensis* (Mali, West Africa) (Latoxan, France)	2.5	0.39 (0.25-0.61)	78.95 (63.80-97.69)	0.309 (0.198-0.483)	0.185
7	*Dendroaspis viridis* (Ghana, West Africa) (Latoxan, France)	1.5	0.15 (0.13-0.17)	139.56 (104.98-185.53)	0.039 (0.034-0.044)	0.013
8	*Dendroaspis polylepis* (Kenya, East Africa) (Latoxan, France)	2.5	0.28 (0.16-0.51)	152.63 (136.44-170.74)	0.105 (0.060-0.192)	0.063
9	*Dendroaspis angusticeps* (Tanzania, East Africa) (Latoxan, France)	1.5	1.53 (1.36-1.71)	>200	Not effective	—
10	*Micrurus nigrocinctus* (Costa Rica) (Wild caught specimens)	2.5	0.51 (0.45-0.58)	139.56 (104.98-185.53)	0.201 (0.177-0.229)	0.121

LD_50_: Median lethal dose; ED_50_: Median effective dose; ER_50_: Median effective ratio.

^a^Median lethal dose was defined as the dose of venom (µg/g) at which 50% of mice died. The 95% confidence limits are included in parenthesis.

^b^Median effective dose was defined as the dose of antivenom (µl) at which 50% of mice survived. The 95% confidence limits are included in parenthesis.

^c^Median effective ratio was defined as the ratio of venom (mg) to antiserum (ml) at which 50% of mice survived.

^d^Potency, P was defined as the neutralization potency of the antivenom (mg venom/ml antiserum) at which the amount of venom (mg) was completely neutralized by one ml of antivenom.

^@^Tan *et al.* (2015)^66^

^#^Tan *et al.* (2016)^20^

^&^Tan *et al.* (2016)^21^.

**Table 2:** Profiles of major lethal toxins of 10 neurotoxic venoms from four different continents.

#	Elapid venoms (locality/source)	Major Lethal Toxins (% of total venom proteins) (numbers of proteoforms reported are shown in brackets)														Ref.
		3FTx									Phospholipase A_2_			KSPI/dendrotoxin	SVMP	
		SNTX	LNTX	CTX	κ-BTX	Mambalgin	Aminergic toxin	Mambin	L-type calcium blocker	Ach-esterase inhibitor	Acidic	Basic	Neutral			
1	*Laticauda colubrina* (Bali, Indonesia) (Ven. Supplies, Australia)	16.9 (2)	48.9 (3)	0.3 (1)	—	—	—	—	—	—	<0.1 (1)	33.2 (4)	—	—	—	^66^
2	*Hydrophis schistosus* (Penang, Malaysia) (Wild caught specimens)	55.8 (1)	14.7 (3)	—	—	—	—	—	—	—	6.1 (1)	21.4 (3)	—	—	0.5 (1)	^20^
3	*Notechis scutatus* (southern Australia) (Ven. Supplies, Australia)	1.7 (1)	4.0 (2)	—	—	—	—	—	—	—	37.3 (8)	32.4 (9)	4.8 (1)	6.9 (3)	—	^21^
4	*Oxyuranus scutellatus* (Australia) (Ven. Supplies, Australia)	1.5% (1)	—	—	—	—	—	—	—	—	45.7 (10)	33.7 (2)	—	7.8 (1)	5.2 (4)	^17^
5	*Pseudechis australis* ^#^ (Australia) (Ven. Supplies, Australia)	—	—	—	—	—	—	—	—	—	✔ (17) (Undifferentiated			✔ (1)	✔ (5)	^15^
6	*Naja senegalensis* ^&^ (Mali, West Africa) (Latoxan, France)	—	—	—	—	—	—	—	—	—	—	—	—	—	—	N.A.
7	*Dendroaspis viridis* (Ghana, West Africa) (Latoxan, France)	13.1 (2)	0.9 (1)	—	—	<0.1 (1)	0.5 (1)	2.1 (1)	7.7 (1)	—	—	—	—	5.6 (7)	—	^24^
8	*Dendroaspis polylepis* (Kenya, South Africa) (Latoxan, France)	3.7 (1)	13.2 (1)	—	—	1.4 (2)	<0.1 (1)	—	2.9 (1)	—	<0.1 (1)	—	—	61.1 (4)	3.2 (14)	^25^
9	*Dendroaspis angusticeps* (Tanzania, East Africa) (Latoxan, France)	—	—	—	—	3.0 (1)	12.6 (3)	6.2 (3)	<0.1 (1)	8.4 (3)	—	—	—	16.3 (5)	6.7 (16)	^23^
10	*Micrurus nigrocinctus* (Costa Rica) (Wild caught specimens)	14.5 (1)	7.3 (1)	—	4.7 (1)	—	—	—	—	—	5.2 (2)	—	~10.0 (3)	—	4.3 (3)	^67^

Abbreviations: Ven. Supplies: Venom Supplies; 3FTx: three-finger toxins; SNTX: short neurotoxin; LNTX: long neurotoxin; CTX: cytotoxin/cardiotoxin; κ-BTX: kappa-bungarotoxin; KSPI: Kunitz-type serine protease inhibitor; SVMP: snake venom metalloproteinase; N.A.: Not available

^&^Proteomic data is not available

^#^Quantitative data is not available.

From the median lethal dose (LD_50_) results, the coastal taipan (*O. scutellatus*) was the most lethal (LD_50_ = 0.03 µg/g) among the 10 venoms, while the least toxic was *D. angusticeps* venom (Tanzania) with LD_50_ of 1.53 µg/g (Table 1).

Of the ten venoms studied, nine of them, including those from the two sea snakes, the Central American coral snake and the Australian snakes were cross-neutralized, and so were those of two African mambas (*D. viridis* and *D. polylepis*) and one African cobra (*N. senegalensis*). Only the green mamba (*D. angusticeps*) venom was not neutralized by the pan-specific antiserum. The antiserum most effectively neutralized the venom of *N. senegalensis* with a Potency (P) of 0.185 mg venom/ml antiserum, followed by the venoms of *M. nigrocinctus*, the two African mambas and the three Australian elapids. The P value of antiserum against the sea krait (*L. colubrina*) venom underscored that it was still capable of neutralization. Thus the results showed that 9 out of ten neurotoxic venoms were neutralized by the pan-specific antiserum; only the venom of *D. angusticeps* was not neutralized even with 200 µl of the antiserum, a dose that was the maximum volume permissible for bolus intravenous injection into mice.

It is relevant to analyse the neutralization results *vis-à-vis* the proteomic profiles previously reported for these venoms. Table 2 depicts the major toxic components described for these venoms, with the exception of *N. senegalensis*, whose venom proteome has not been characterized. The proteomics toxin profiles show the major toxic (lethal) components of each of these 10 venoms. Seven of them contain short (Type I) and long (Type II) α-neurotoxins, which belong to the three-finger toxin family (3FTx). The α-neurotoxins in these seven venoms are highly toxic and could lead to neuromuscular paralysis and death.

**Figure 1:**
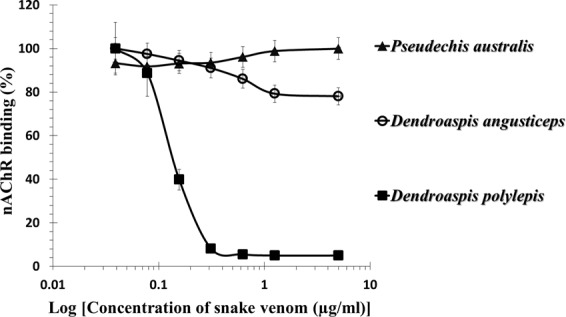
Inhibition of nAchR binding to NK3 coated plate by various venoms: –▲–▲–▲– *Pseudechis australis*; –○–○–○– *Dendroaspis angusticeps*; –■–■–■– *Dendroaspis polylepis*. Each points was the mean ± SEM of 3 separate determinations.

Neurotoxicity caused by *P. australis* venom has been demonstrated and it was more potent to diapsids than to synapsids^14^. Moreover, a short- and a long α-neurotoxins had been reported from *P. australis* venom (UniProt database entries: P25497 and P14612, respectively). The toxins were present at very low level that probably explained its non-detection in the proteomic study^15^, and in our *in vitro* assay based on *T. californica* nAChR binding (Fig. 1). However, the α-neurotoxins were highly lethal to mice (LD_50_ < 0.1 µg/g)^16^, and along with the myotoxic and anticoagulant PLA_2_s probably contribute to the venom lethality in mice, being therefore possible targets of cross-neutralization by the pan-specific antiserum.

All nine venoms with available proteomic information contained phospholipases A_2_ (PLA_2_s), some of which are basic PLA_2_ that contribute to presynaptic neurotoxicity in *O. scutellatus* (known as taipoxin)^17^ and *N. scutatus* (known as notexin)^18^ venoms, and hemolytic, anticoagulant as well as myotoxic activities in *P. australis* venom^19^. Besides, the myotoxic PLA_2_ was also found abundantly in the sea snake (*H. schistosus*) venom, which causes rhabdomyolysis and nephrotoxicity in envenomation^20^.

Both lethal α-neurotoxins and presynaptic PLA_2_s are present in the venoms of *H. schistosus*^20^ and *N. scutatus*^21,22^. In addition to α- and β-neurotoxins, procoagulant serine proteases in the venoms of Australian elapids (*O. scutellatus* and *N. scutatus*) can cause hemostatic alterations which may lead to bleeding or thrombosis^17,23^, although it is likely that neurotoxins are the main culprits of lethality in these venoms. The lethal toxins present in the nine venoms were presumably neutralized by the pan-specific antiserum, as evidenced by neutralization results.

It should be mentioned that the pan-specific antivenom was prepared with the aim of neutralizing lethal neurotoxins (mainly α- and β-neurotoxins) and not against the high molecular weight enzymes (e.g. prothrombin activators) which were filtered out during the preparation of immunogens. As such, the pan specific antiserum was not tested for neutralization of these activities associated with high molecular mass components.

Regarding the three mamba venoms, α-neurotoxins are present in *D. viridis* and *D. polylepis*^24,25^. In addition, fasciculins, members of the 3FTx family which induce fasciculations by inhibiting acetylcholinesterase, were found in *D. viridis* and *D. angusticeps* venoms^24^. Dendrotoxins, which have homology to Kunitz-type proteinase inhibitors and block voltage-dependent potassium channels, are typical of mamba venoms, with highest concentration in the venom of *D. polylepis*^24^. Both dendrotoxins and fasciculins were probably not neutralized by the pan-specific antiserum since these toxins are not present in the immunogen mix. α-Neurotoxins are the most lethal toxins of *D. polylepis* venom, with dendrotoxins playing a minor role in lethality^25^. This explains why the lethality of this venom was neutralized by the pan-specific antiserum even though the dendrotoxins were unlikely neutralized.

*D. angusticeps* venom was the only one not neutralized by the pan-specific antiserum. From its proteome, fourty-two different proteins were detected, among which 3FTxs were the most abundant, followed by the Kunitz-type proteinase inhibitor family. However, no α-neurotoxin was identified in the venom^23^ which is in agreement with an *in vitro* potency assay based on nAChR binding^26^ (Fig. 1). None of the venom HPLC fractions was lethal to mice at the doses tested. Thus, it was proposed that the lethality of the venom was due to the synergistic action of various components, such as fasciculins and dendrotoxins, and probably other synergistically-acting toxins^23^. It is not surprising that the pan-specific antiserum did not neutralize the lethal effects of the venom since the toxins of the venom were not present in the immunogen mix, and simultaneous neutralization of various synergistic acting toxins are required in order to neutralize the lethality of the venom.

Proteomics data indicate that α-neurotoxins in *N. nigricollis* venom was only 0.4% of the total HPLC separated proteins, with higher amounts of cytotoxins (72.8%) and PLA_2_s (21.9%)^27^. The antiserum most likely contains antibodies against these components in this spitting cobra venom probably due to the presence of similar toxins in the venoms used in the immunizing mix.

In the case of *N. senegalensis* venom, no proteomics data are available. However, clinical cases are associated with neuromuscular paralysis and respiratory failure^28^, suggesting that α- neurotoxins are likely to play a key role in the overall toxicity*.* This venom was effectively neutralized by the pan-specific antiserum, underscoring that these lethal toxins were immunorecognized by the antibodies. Nevertheless, caution should be exerted when extrapolating data from mouse experiments to the human situation when studying venom-induced neurotoxicity^29^.

Table 3 shows the proteomics toxin profiles of the 16 heterologous venoms previously shown to be neutralized by the pan-specific antiserum. Ten of them are from species of the genus *Naja*, characterized by the presence of short (Type I) and long (Type II) α-neurotoxins and cytotoxins. Venoms from species of *Bungarus*, e.g. *B. candidus* and *B. multicinctus*, contain both pre-synaptic β- and post-synaptic α-neurotoxins^30,31^. In the case of *Ophiophagus hannah*, its venom contains α-neurotoxins but not β-neurotoxins^32^. These toxins, except for cytotoxins, are highly lethal in mice and are known to be the cause of death in elapid envenomations. On the basis of our observations, they were likely neutralized by the antibodies in the pan-specific antiserum.

**Table 3:** Profiles of major lethal toxins of 16 heterologous elapid venoms tested in previous study (Ratanabanangkoon *et al*., 2016).

No	Elapid venoms (locality/source)	Major Lethal Toxins (numbers of proteoforms reported are shown in parentheses)											Ref.
		3FTx (%)					Phospholipase A_2_ (%)				Kunitz	SVMP	
		SNTX	LNTX	CTX	α-BTX	κ-BTX	Acidic	Basic	Neutral	β-BTX			
1	*Naja oxiana*^&^ (Pakistan) (Wild caught specimens)	—	—	—	—	—	—	—	—	—	—	—	N.A.
2	*Naja sumatrana* (Seremban, Malaysia) (Wild caught specimens)	3.5 (2)	12.1 (2)	44.2 (6)	—	—	5.0 (3)	—	27.3 (1)	—	—	✓	^68^
3	*Naja siamensis* (Thailand) (Latoxan, France)	~4.7 (3)	~22.6 (2)	33.8 (14)	—	—	15.3 (15) (Undifferentiated)			—	—	1.45 (4)	^69^
4	*Naja naja*^@^ (India) (Latoxan, France)	1.6 (3)	2.1 (1)	69.3 (10)	—	—	21.4 (1)	—	—	—	0.1 (1)	0.9 (1)	^70^
5	*Naja naja*^@^ (Sri Lanka) (Wild caught specimens)	1.5 (3)	4.7 (2)	71.6 (10)	—	—	13.9 (1)	—	—	—	0.3 (1)	0.9 (1)	^70^
6	*Naja naja* (Pakistan) (Dr Naeem Qairaishi, Sindh) (Wild caught specimens)	4.7 (2)	21.6 (3)	46.9 (5)	—	—	10.6 (3)	1.4 (1)	2.3 (1)	—	0.9 (1)	1.5 (3)	^71^
7	*Naja haje*^@#^ (Morocco) (serpentarium of Pasteur Institute of Morocco)	✓			—	—	—		—	—	—	—	^79^
		(3)	(5)	(17)			(3)	(1)					
8	*Naja nigricollis*^#^ (Cameroon) (Latoxan, France)	✓	—	✓	—	—	✓	✓	—	—	—	✓	^27^
9	*Bungarus caeruleus* (Pakistan) (Wild caught specimens)	—	—	—	—	15.6 (2)	59.9 (5)	—	—	4.6 (4)	4.4 (3)	1.3 (1)	^72^
10	*Bungarus caeruleus* (Sri Lanka) (Serpentarium, University of Colombo)	Chromatographic profile of Sri Lankan *B. caeruleus* did not exhibit significant variation compared to Pakistani *B. caeruleus*											
11	*Bungarus caeruleus* (India) (Latoxan, France)	Chromatographic profile of Indian *B. caeruleus* did not exhibit significant variation compared to Pakistani *B. caeruleus*											
12	^*Bungarus fasciatus*^ (Thailand) (Queen Saovabha Memorial Institute)	1.6 (1)	—	—	—	—	11.5 (4)	54.2 (9)	0.01 (1)	—	21.3 (3)	2.4 (3)	^80^
13	*Bungarus sindanus* (Pakistan) (Wild caught specimens)	0.5 (1)	—	—	—	6.2 (3)	16.8 (7)	—	—	15.8 (7)	13.3 (3)	0.5 (3)	^73^
14	*Ophiophagus hannah* (Seremban, Malaysia) (Wild caught specimens)	7.5 (2)	26.7 (6)	0.5 (1)	—	—	4.0 (1)	—	—	—	1.0 (1)	24.4 (12)	^32^
15	*Naja melanoleuca*^@^ (Uganda) (Latoxan, France)	7.3 (1)	~13.4 (4)	25.2 (2)	—	—	~4.3 (3)	~1.4 (1)	~7.3 (1)	—	3.8 (1)	~9.2 (8)	^74^
16	*Naja nubiae*^@^ (North Africa) (Latoxan, France)	12.6 (2)	—	58.3 (10)	—	—	26.4 (1)	(1)	—	—	—	2.6 (1)	^27^

Abbreviations: 3FTx: three-finger toxins; SNTX: short (Type I) neurotoxin; LNTX: long (Type II) neurotoxin; CTX: cytotoxin/cardiotoxin; α-BTX: alpha-bungarotoxin; κ-BTX: kappa-bungarotoxin; β-BTX: beta-bungarotoxin; KSPI: Kunitz-type serine protease inhibitor; SVMP: snake venom metalloproteinase; N.A.: Not available.

^&^Proteomic data is not available.

^#^Quantitative data is not available.

^@^Venom source different from the species tested in the previous study^11^.

Thus, the pan-specific antiserum neutralized most, if not all, the potentially lethal toxins in the 25 heterologous neurotoxic venoms tested, hence stressing the value of using fractions enriched with various lethal toxins of several venoms in the immunization process. Another alternative proposed to raise an antiserum of high neutralizing coverage against neurotoxic venoms includes the use of recombinant consensus short α-neurotoxins as immunogen^33^. In this case, however, the antiserum is not effective in the neutralization of presynaptic β-neurotoxins, and recombinant consensus PLA_2_ neurotoxins would need to be added to the immunizing mix to neutralize venoms whose toxicity is driven by β-neurotoxins.

The preparation of this wide para-specific antiserum involved immunization of horses with 12 toxin fractions/venoms of 6 species of selected cobras (*Naja spp*.) and kraits (*Bungarus spp*.) inhabiting various countries of Asia. Table 4 presents the toxin profiles of these 12 venoms. These elapid venoms contain a diverse set of toxins, such as short and long α-neurotoxins, β-neurotoxins and cytotoxins as the major lethal toxins. Table 4 shows the number of isoforms of each type of these toxins in these 12 venoms. Altogether, there are about 23 isoforms of short (Type I) α-neurotoxins, 17 isoforms of long (Type II) α-neurotoxins and about 15 isoforms of PLA_2_ β-neurotoxins which are exposed to the horses. These large numbers of toxin isoforms are likely to contain numerous epitopes of these lethal toxins. It is therefore conceivable that the immune system of the horses generated a diverse set of paratopes against all the isoforms of these lethal toxins, hence explaining the ability of the antiserum to neutralize the lethal activity of 36 neurotoxic elapid venoms (25 heterologous and 11 homologous venoms) from 10 snake genera.

**Table 4:** Toxin profiles of 12 elapid venoms used as immunogens for production of horse pan-specific antisera^11^.

No	Elapid venoms (locality/source)	Potential Lethal Toxins (numbers of proteoforms detected are shown in brackets)											Ref.
		3FTx (%)					Phospholipase A_2_ (%)				Kunitz	SVMP	
		SNTX	LNTX	CTX	α-BTX	κ-BTX	Acidic	Basic	Neutral	β-BTX			
***Naja***													
1	*Naja kaouthia* (Thailand) (QSMI, Thailand)	7.7 (2)	33.3 (1)	27.6 (6)	—	—	12.2 (2)	—	—	—	—	2.5 (3)	^44^
2	*Naja kaouthia* (Malaysia) (Wild caught specimens)	4.2 (3)	3.9 (1)	45.7 (6)	—	—	23.5 (4)	—	—	—	0.5 (1)	3.3 (4)	^44^
3	*Naja kaouthia* (Vietnam) (Dr. Trinh Xuan Kiem) (Wild caught specimens)	9.2 (3)	—	44.9 (6)	—	—	17.4 (2)	—	—	—	<0.1 (1)	1.6 (3)	^44^
4	*Naja philippinensis* (The Philippines) (Latoxan, France)	44.6(4)	—	21.3 (8	—	—	18.7 (4)	0.5 (1)	3.7 (1)	—	—	3.9 (4)	^75^
5	*Naja sputatrix* (Indonesia) (Latoxan, France)	7.9 (5)	0.5 (3)	48.1 (11)	—	—	23.1 (6)	5.9 (1)	2.3 (1)	—	0.2 (1)	1.3 (10)	^76^
6	*Naja atra*^@^ (China) (Wild caught specimens)	11.3(2)	—	65.3 (4)	—	—	12.2 (2)	—	—	—	—	1.5 (2)	^31^
7	*Naja atra*@ (Western Taiwan) (Wild caught specimens)	23.5 (Undifferentiated)		52.9 (6)	—	—	16.8 (1)		—	—	—	1.7 (2)	^77^
		(2)	(1)										
***Bungarus***													
8	*Bungarus multicinctus*^@^ (China) (Wild caught specimens)	—	—	—	6.% (1)	2.4 (3)	3.3 (1)		—	58.4 (4)	0.5 (2)	0.1 (2)	^31^
9	*Bungarus multicinctus*^# @^ (Taiwan) (Wild caught specimens)	✓ (1)	✓ (7)	—	—	—	✓ (4) (Undifferentiated)				—	✓ (1)	^78^
10	*Bungarus candidus*^&^ (Northeast Thailand) (QSMI, Thailand)	—	—	—	—	—	—	—	—	—	—	—	N.A.
11	*Bungarus candidus*^#^ (Southern Thailand) (QSMI, Thailand)	✓ (1)	✓ (3)	✓ (2)	✓ (6)	✓ (2)	✓ (3)	✓ (4)	—	—	✓ (3)	✓ (2)	^30^
12	*Bungarus candidus*^#^ (Indonesia) (Biopharma, Bandung), (Wild caught specimens)	—	✓ (1)	✓ (1)	—	✓ (2)	—	✓ (2)	—	—	✓ (3)	—	^30^

Abbreviations: 3FTx: three-finger toxins; SNTX: short neurotoxin; LNTX: long neurotoxin; CTX: cytotoxin/cardiotoxin; α-BTX: alpha-bungarotoxin; κ-BTX: kappa-bungarotoxin; β-BTX: beta-bungarotoxin; KSPI: Kunitz-type serine protease inhibitor; SVMP: snake venom metalloproteinase; N.A.: Not available.

^&^Proteomic data is not available.

^#^Quantitative data is not available.

^@^Venom source different from the species tested in the previous study^11^.

### The bases for the wide specificity

It is evident that the experimental antiserum showed very wide para-specificity against numerous neurotoxic venoms. This antiserum was produced by horses which were exposed to epitopes of widely diverse toxins of numerous neurotoxic venoms, which is termed ‘diverse toxin repertoire’. The following considerations may form the bases for explaining this phenomenon:

- The most lethal components in the majority of these neurotoxic venoms are the post synaptically-acting α–neurotoxins (Tables 2, 3 and 4). For some venoms, e.g. *Bungarus spp. and O. scutellatus*, highly lethal β-neurotoxins are also relevant to human envenomation^22,34–38^. For simplicity, this discussion will be confined to the case of α–neurotoxins, which constitute the main lethal factors of many, but not all, elapid venoms^39^.
  α–neurotoxins are small polypeptides of about 61–62 amino acids with 4 disulfide linkages (short α-neurotoxins) or 66–75 amino acids with 5 disulfide linkages (long α-neurotoxins)^35^. They adopt a planar structure similar to a 3-finger configuration and are referred to as three-finger toxins (3FTxs)^40^. The amino acid sequences of the over 400 α–neurotoxins from snakes have been described; the top 100 of these sequences share about 66.8% identity^41^. All of these α–neurotoxins bind specifically and strongly to the α–subunits of nAchR at the motor endplate in the neuromuscular junction^40,42,43^. Thus all these toxins share structural and functional homology.
- Each elapid venom may contain several α–neurotoxins (short, long, dimers, precursors, etc.) that show sequence variations^44^. Most of the venoms contain 1–5 α-neurotoxin isoforms (Table 4); therefore, the total number of α-neurotoxin isoforms in the immunogen mixture may reach several dozens. These numerous α-neurotoxin isoforms together contain a large number of toxin epitopes to which the horses were exposed to, with a vast number of antibody paratopes generated against these epitopes. Hence, the diversity of α-neurotoxin isoforms is the strength in this immunization strategy, as it contributes to generate a wide repertoire of antibodies.
- Given their small molecular size and constraint to form a biologically active conformation, it is likely that each α–neurotoxin contains a relatively small number of dominant epitopes on its surface, as shown for venoms of *Dendroaspis spp*. using a high-throughput microarray analysis^45^, with each epitope covering an area of 6–7 amino acid residues^46^. Some of the epitopes from homologous toxins are conserved for structural and functional reasons. Because of the high sequence identity, some of these epitopes are expected to be structurally similar, though not identical, and thus explain some degree of immunochemical cross reactivity of antisera^47–49^.
- In the case of monospecific polyclonal antisera raised against a single neurotoxic venom, they are likely to contain high affinity antibodies against the α–neurotoxins of the homologous venom. Some of these antibodies may cross react with heterologous α–neurotoxins due to epitope structural similarity. Being heterologous, these cross reacting antibodies in monospecific antisera are likely to have lower affinity/avidity^50^ and by themselves may not bind effectively and neutralize the heterologous α–neurotoxins. This could be a reason for the low cross-neutralization of monospecific antivenoms usually observed^5^.
- The ‘diverse toxin repertoire’ immunization strategy using a large number of diverse α-neurotoxin epitopes to immunize the horses^11^ is likely to result in the production of numerous paratopes in the antibodies against these epitopes which might be shared with other heterologous α–neurotoxins, hence contributing to its broad specificity. Moreover, it has been shown that a single antibody could adopt different conformations of its paratope to bind different epitopes, thus enhancing its antigenic coverage^51^.
- The more diverse the paratopes of the antisera antibodies, the better chance for some of them to interact with the epitopes of the heterologous α-neurotoxins. These interactions, albeit with lower affinity should, through cross-linking and lattice formation^52^, result in antisera with higher avidity leading to more effective neutralization of diverse heterologous neurotoxic venoms^53^. Similar situations may occur with the highly lethal presynaptic β-neurotoxins^37^ and other types of venom toxins when using a variety of venoms in the immunizing mixture.
- The large pool of diverse antibody paratopes makes it likely that high affinity antibodies are present to interact effectively with a variety of α-neurotoxins. This is crucial because, due to steric hindrance, no more than two antibody molecules can interact simultaneously with one toxin molecule (Supplementary Fig. 1).

Whether or not the proposed bases for the wide para-specificity of our antiserum are correct, the results of these studies show that this is the widest cross-neutralizing antiserum ever reported against neurotoxic snake venoms from wide geographical distribution. Our results represent a proof of concept that an antiserum with wide spectrum of cross-neutralization against elapid venoms can be raised.

In this connection, it would be interesting to apply the ‘diverse toxin repertoire’ immunization strategy to prepare pan-specific antisera against other snake venoms in which different toxicological effects predominate, such as hemorrhagic and procoagulant venoms in sub-Saharan Africa^23,24^. The genus-wide analysis of venom composition and toxicity of these venoms to identify the lethal toxins^24^ followed by use of the combined toxin fractions to immunize horses, is likely to result in widely para-specific antiserum against these snake venoms.

### Further possible improvements of the pan-specific antisera against neurotoxic venoms

As shown in Tables 1, 2 and 3, the antiserum could neutralize lethality of 25 heterologous venoms, but its neutralizing potency against some of them is rather low. This becomes a problem especially when dealing with species that inject a large volume of venom in a bite. However, the potency of the antiserum can be improved by a concentration process during plasma fractionation. For example, the antivenom produced from horse sera against the recombinant short chain α-neurotoxins was fractionated by caprylic acid precipitation and used at 50 mg/ml^33^; hence, the protein in this antivenom most likely contained only IgG and IgG_T_. Since horse hyperimmune sera have an average protein concentration at 62.75 ± 0.33 mg/ml, and IgG + IgGT represent about 37% of the total serum protein^54^, the antibody in the antivenom prepared by de la Rosa *et al*.^33^ was about 2.01 fold concentrated as compared to the crude horse serum. Also, the commercial antivenoms produced by the Thai Red Cross in the form of F(ab’)_2_ resulted from about three fold concentrates of serum IgG. After such concentration process, the present horse pan-specific antiserum could have higher neutralizing activity against the lethality of many neurotoxic venoms. This may not only increase the potency against the venoms tested, but also provide neutralization of additional elapid venoms.

There is a growing interest in the development of recombinant antivenoms^55,56^. This involves, for example, the preparation of animal- or human-derived monoclonal antibodies against the lethal components of venoms. Proofs of concept of this strategy have been published^57,58^. One major requirement of this approach is that the major lethal toxin(s) of the venom must be identified and used for antibody selection. When more than one toxin is relevant in a particular venom, there is a need to generate additional antibodies for a successful neutralization. Since these antibodies are produced against one or few toxins, a challenging issue for this strategy is to ensure the neutralization of heterologous toxins present in other venoms. Such problem is overcome by the present ‘diverse toxin repertoire’ immunization approach by including the toxin/venom fractions of a variety of venoms in the immunogen mix, thus ensuring cross-reactivity, as described in this work.

It should be possible to further increase the para-specificity of the antiserum by including additional venom toxin fractions in the immunization mix. For example, inclusion of toxin fractions of some African mamba venoms (*D. angusticeps* and *D. viridis*) and some American coral snakes (e.g., *Micrurus spp.)* could increase the neutralizing scope of the antiserum. Noteworthy, the amount of toxin fraction of each venom used in the immunization was just a few micrograms (12 µg of each venom in the primary immunization), and the total protein amount of such an immunization mix would still be less than one mg^11^. This amount of venom toxins, once emulsified in Freund’s oil adjuvants, does not cause major harm to the horses^59^. By carefully selecting the venoms and fractions to be added to the immunizing mix it should be possible to expand the scope of coverage of neurotoxic venoms, ideally to neutralize the most important elapid venoms in the world.

## Conclusion

Snakebite envenomation is a WHO classified category A Neglected Tropical Diseases, i.e. a disease of highest importance. Consequently, WHO has launched a global strategy with the aim of reducing the disease burden of snakebite envenomation by 50% by the year 2030^60^. One of the four pillars of this strategy is to ensure safe and effective treatments, particularly referring to antivenoms, which represent the only scientifically-validated therapy for these envenomings. As shown in this work, a pan-specific antivenom against neurotoxic venoms would be a powerful therapeutic tool to save lives of people suffering these envenomings in different parts of the world, by neutralizing a wide spectrum of neurotoxic snake venoms which otherwise require region- or species-specific antivenoms for treatment. We showed that the production of such an antivenom prototype is feasible and practical through exposing horses to a novel ‘diverse toxin repertoire’ of 12 neurotoxic snake toxins/venoms. The antiserum exhibited a wide para-specificity by neutralizing at least 36 neurotoxic venoms of snakes of 10 genera from four continents. The pool of diverse toxin antigens in the immunogen mix enabled the production of diverse antibody paratopes, which facilitate the interaction of the antibodies with the epitopes of various neurotoxins from homologous as well as heterologous snake venoms. With further optimization on the immunogen mix, the ‘diverse toxin repertoire’ immunization strategy could result in antisera with even broader para-specificity, ideally to cover most or all medically important neurotoxic venoms worldwide.

## Materials and Methods

### Venoms and antisera

*Dendroaspis polylepis, D. angusticeps, D. viridis* and *Naja senegalensis* venoms were obtained from Latoxan (Valence, France); *Laticauda colubrina, Pseudechis australis, Oxyuranus scutellatus* and *Notechis scutatus* venoms were obtained from Venom Supplies Pty Ltd (Australia). *Hydrophis schistosus* venom was provided by Dr. CH Tan, and *Micrurus nigrocinctus* venom was provided by Prof. José María Gutiérrez. These two venoms were obtained from several specimens kept in captivity (*M. nigrocinctus*) or captured wild (*H. schistosus*).

*Naja kaouthia* (Thailand) principal post-synaptic neurotoxin 3 (NK3) was purified as described by Karlsson *et al*.^61^. The pan-specific antiserum used in the present study was from the same batch as that obtained from horses immunized with mixtures of venoms and venom fractions^11^ using the protocol briefly described below.

### Chemicals

All chemicals and biochemical were from Sigma Chemical Co. (St Louis, Missouri, USA) unless otherwise stated.

### Animal care and experimentation

Experiments carried out in horses regarding care, bleeding and immunization were approved by the Animal Care and Use Committee of the Faculty of Veterinary Science, Mahidol University, Protocol and clearance no.MUVS-2012-69 in accordance with the Guidelines of the National Research Council of Thailand.

Albino mice (male ICR strain, 20–30 g) were supplied by the Animal Experimental Unit (AEU), Faculty of Medicine, University of Malaya, and handled according to the Council for International Organization of Medical Sciences (CIOMS) guideline on animal experimentation^62^. The animal use was approved by the Institutional Animal Care and Use Committee (IACUC) of the University of Malaya (Reference number: 2018-211218/MOL/R/TKY).

### Preparation of the pan-specific antiserum

Preparation of the pan-specific antiserum was described previously^11^. Briefly, horses were immunized with a mixture of toxin fraction/venoms of 12 elapids mostly of *Naja spp*. and *Bungarus spp*. inhabiting different geographical locations of Asia^11^. The toxin fractions of *Naja spp*. venoms were prepared by ultrafiltration of each venom solution (prepared in 100 mM ammonium acetate, pH 5.0, 1 mg/ml) through 30 kDa molecular weight cut off (MWCO) ultrafiltration membranes while those of the *Bungarus spp*. venoms were filtered through 50 kDa MWCO filters. The toxin fractions (12 μg from each venom) were combined and mixed with (1:1 v/v) and emulsified in Complete Freund Adjuvant. The primary immunization was carried out using the ‘low dose, low volume multi-site’ immunization protocol^59,63^ under which the immunogen was injected subcutaneously around the horse’s neck at 20 sites in a volume of 0.1 ml/site. After several booster immunizations at 2 weeks intervals, the horses were bled and the sera prepared by centrifugation of the clotted blood at 800 × g for 15 minutes. The sera were kept at −20 °C.

### Estimation of α-neurotoxins present in neurotoxic venoms by their binding to solubilized Torpedo californica nicotinic acetylcholine receptor (nAChR)

The presence of α-neurotoxins in venoms was estimated by the venom-mediated inhibition of the binding of purified nAChR to immobilized elapid post-synaptic neurotoxins, as described previously^26^. The basic procedure of the assay is to incubate (25 °C, 60 min) varying amounts (0.03–10 μg/ml) of a tested neurotoxic venom with a pre-determined optimal amount (0.707 µg/ml) of soluble nAChR purified from *T. californica* electroplax. After incubation, the excess nAChR was estimated by adding the mixture to microtiter wells coated with 15 µg/ml of purified NK3. The amount of nAChR bound to the immobilized NK3 in the wells was estimated by adding rat anti-nAChR serum at 1:1600 dilution followed by a 1:4500 dilution of goat anti-rat IgG-enzyme conjugated HRP and enzyme substrate. In the absence of the tested neurotoxic venom, the binding of nAChR was considered as 100%. If the tested neurotoxic venom contained α-neurotoxin which could specifically interact with nAChR, the percent binding of the receptor to the NK3 immobilized plate is reduced and can be calculated using the following formula:

$${\rm{ \% }}\,{\rm{n}}{\rm{A}}{\rm{C}}{\rm{h}}{\rm{R}}\,{\rm{b}}{\rm{i}}{\rm{n}}{\rm{d}}{\rm{i}}{\rm{n}}{\rm{g}}=\frac{({\rm{O}}{\rm{D}}\,{\rm{S}}{\rm{a}}{\rm{m}}{\rm{p}}{\rm{l}}{\rm{e}}\,-\,{\rm{O}}{\rm{D}}\,{\rm{A}}{\rm{g}}\,{\rm{c}}{\rm{o}}{\rm{n}}{\rm{t}}{\rm{r}}{\rm{o}}{\rm{l}})\times 100}{({\rm{O}}{\rm{D}}\,{\rm{M}}{\rm{a}}{\rm{x}}\,-\,{\rm{O}}{\rm{D}}\,{\rm{c}}{\rm{o}}{\rm{n}}{\rm{t}}{\rm{r}}{\rm{o}}{\rm{l}})}$$

‘OD Max’ represents the binding of optimized nAChR to NK3 immobilized plate without incubation with each crude elapid venoms.

‘OD Ag control’ represents the binding background of optimized nAChR and NK3 immobilized plate.

‘OD Sample’ represents the binding of optimized nAChR to the NK3 immobilized plate after the nAChR was inhibited by α- neurotoxin at various concentrations of crude elapid venoms.

### Determination of venom lethality and antiserum efficacy of neutralization in mice

Venom lethality (median lethal dose, LD_50_) and the median effective doses (ED_50_) of the pan-specific antiserum against the venoms tested were determined and analyzed as previously reported^11^ and are briefly described below.

#### Determination of LD_50_

The median lethal dose (LD_50_) of a venom was determined by *i.v.* injection of varying doses of the venom into the caudal vein of the mice (20–30 gm, n = 4 per venom dose). The result was obtained at 24 hr after injection and LD_50_ was determined using Probit analysis^64^.

### Lethality neutralization assay

In the lethality neutralization assay, a challenge dose of 5xLD_50_ of a venom was mixed with various doses of the antiserum (diluted in normal saline) and pre-incubated at 37 °C for 30 minutes. The mixture was centrifuged at 10,000 × g for 5 minutes and aliquots of the supernatant, containing 5 LD_50_s of venom, were injected intravenously into the caudal vein of the mice (n = 4 per dose). If the mice did not fully survived 5x LD_50_ of venom dose with 200 µl of antiserum, the challenge dose was reduced to 2.5x LD_50_ and subsequently to 1.5x LD_50_ if needed. In all experiments, the control groups of mice, regardless of whether 5x, 2.5x or 1.5x LD_50_ challenge dose without the use of the antiserum died.

The neutralization potency (P) of the antiserum, defined as the amount of venom completely neutralized per unit volume of antiserum, was expressed as previously described^65^.

## Supplementary information

Supplementary Information.
