# Supplementary figures and images for: A pan-specific antiserum produced by a novel immunization strategy shows a high spectrum of neutralization against neurotoxic snake venoms
Source: Sci Rep. 2020 Jul 9;10:11261. doi: 10.1038/s41598-020-66657-8 (PMC7347863; doi:10.1038/s41598-020-66657-8)

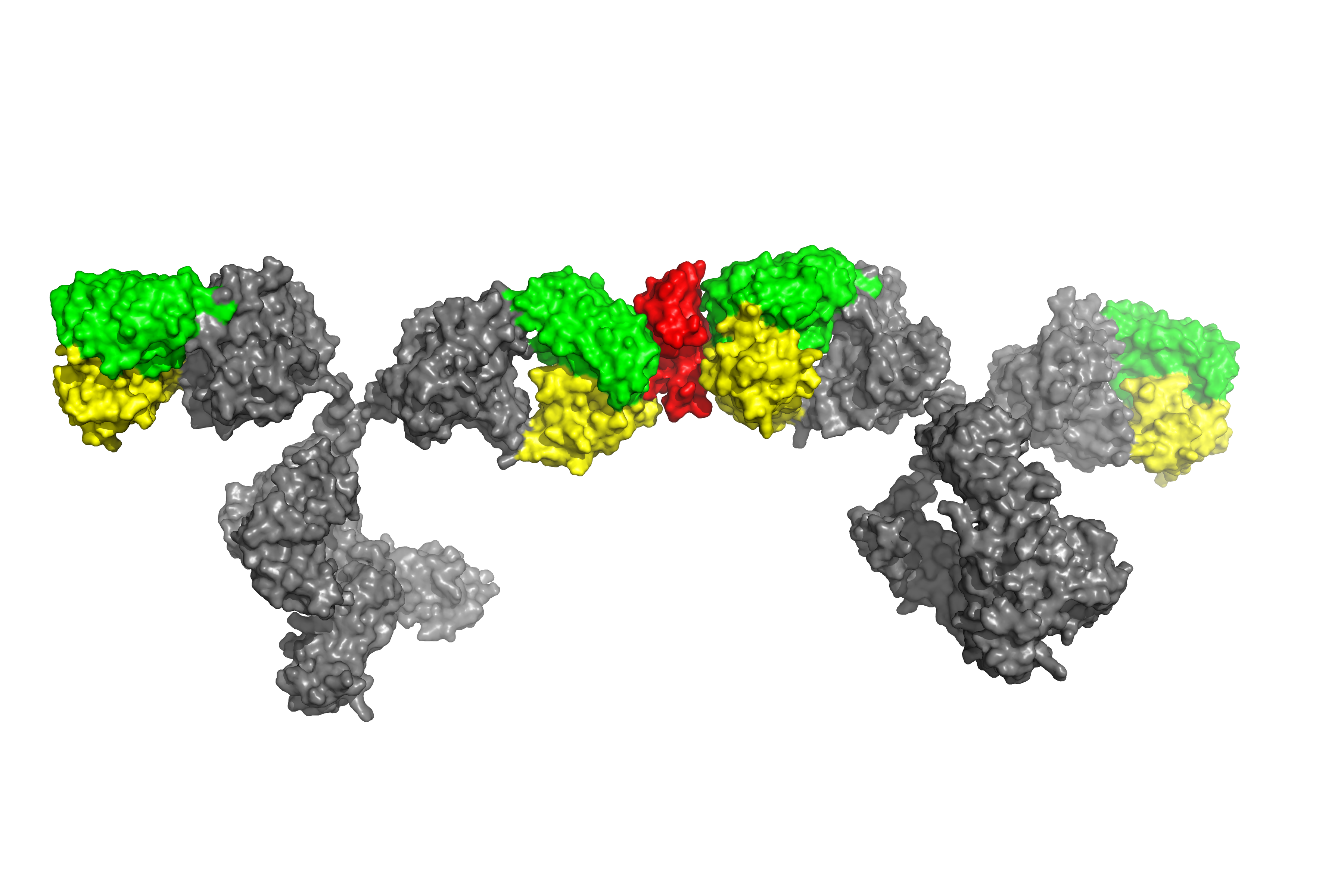

Supplement: Supplementary file 1 — Supplementary Information. [file 41598_2020_66657_MOESM1_ESM.tif]
